# Precision 3D printed meniscus scaffolds to facilitate hMSCs proliferation and chondrogenic differentiation for tissue regeneration

**DOI:** 10.1186/s12951-021-01141-7

**Published:** 2021-12-02

**Authors:** Xingyu Deng, Xiabin Chen, Fang Geng, Xin Tang, Zhenzhen Li, Jie Zhang, Yikai Wang, Fangqian Wang, Na Zheng, Peng Wang, Xiaohua Yu, Shurong Hou, Wei Zhang

**Affiliations:** 1 School of Pharmacy, Hangzhou Normal University, Hangzhou, 311121 Zhejiang China; 2 Medtronic Technology Center, Shanghai, 201114 China; 3 Department of Orthopaedics, The Second Affiliated Hospital of Zhejiang University School of Medicine, Hangzhou, 310009 China; 4 Zhejiang Provincial Key Laboratory of Orthopaedics, Hangzhou, Zhejiang Province China; 5 State Key Laboratory of Chemical Engineering, School of Chemical and Biological Engineering, Zhejiang University, Hangzhou, 310027 China; 6 The State Key Laboratory of Translational Medicine and Innovative Drug Development, Nanjing, 210042 China; 7 Orthopedics Research Institute of Zhejiang University, Hangzhou, 310009 Zhejiang Province China

**Keywords:** Meniscus, Tissue engineering, Scaffold, Chondrogenic differentiation

## Abstract

**Background:**

The poor regenerative capability and structural complexity make the reconstruction of meniscus particularly challenging in clinic. 3D printing of polymer scaffolds holds the promise of precisely constructing complex tissue architecture, however the resultant scaffolds usually lack of sufficient bioactivity to effectively generate new tissue.

**Results:**

Herein, 3D printing-based strategy via the cryo-printing technology was employed to fabricate customized polyurethane (PU) porous scaffolds that mimic native meniscus. In order to enhance scaffold bioactivity for human mesenchymal stem cells (hMSCs) culture, scaffold surface modification through the physical absorption of collagen I and fibronectin (FN) were investigated by cell live/dead staining and cell viability assays. The results indicated that coating with fibronectin outperformed coating with collagen I in promoting multiple-aspect stem cell functions, and fibronectin favors long-term culture required for chondrogenesis on scaffolds. In situ chondrogenic differentiation of hMSCs resulted in a time-dependent upregulation of SOX9 and extracellular matrix (ECM) assessed by qRT-PCR analysis, and enhanced deposition of collagen II and aggrecan confirmed by immunostaining and western blot analysis. Gene expression data also revealed 3D porous scaffolds coupled with surface functionalization greatly facilitated chondrogenesis of hMSCs. In addition, the subcutaneous implantation of 3D porous PU scaffolds on SD rats did not induce local inflammation and integrated well with surrounding tissues, suggesting good in vivo biocompatibility.

**Conclusions:**

Overall, this study presents an approach to fabricate biocompatible meniscus constructs that not only recapitulate the architecture and mechanical property of native meniscus, but also have desired bioactivity for hMSCs culture and cartilage regeneration. The generated 3D meniscus-mimicking scaffolds incorporated with hMSCs offer great promise in tissue engineering strategies for meniscus regeneration.

**Graphical Abstract:**

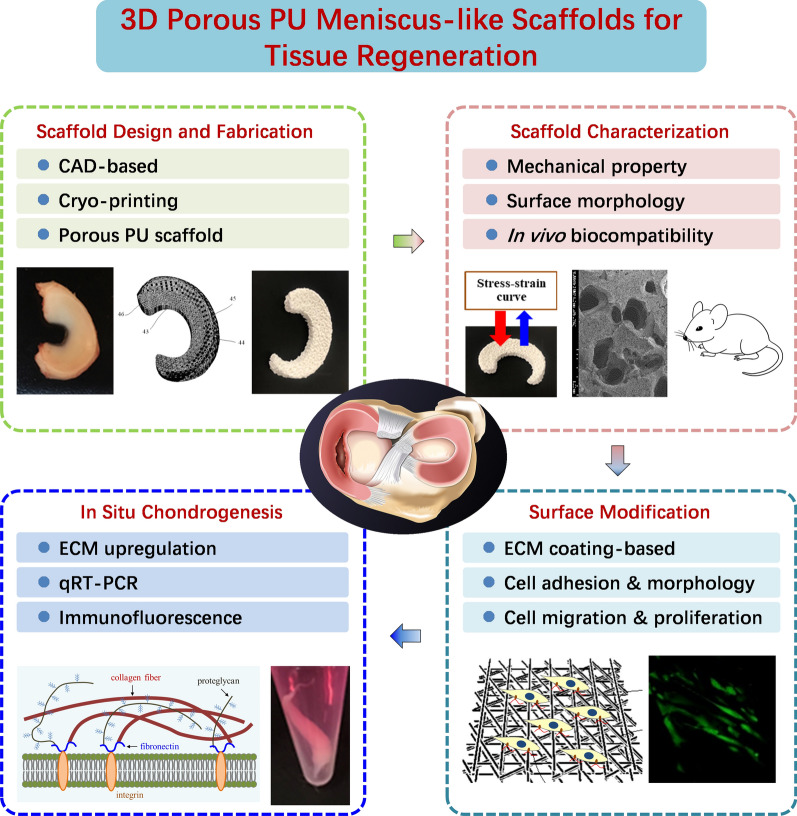

## Background

The menisci in the knee joint are the two pieces of crescent-shaped cartilage between the distal femur and the proximal tibia condyle, which play a critical role in load bearing, load transmission, shock absorption and joint lubrication [1, 2]. Meniscus injury primarily resulted from physical trauma or degenerative process, has become one of the most prevalent and challenging diseases of the knee joint [3, 4]. A torn meniscus causes persistent knee pain, limited mobility, and further degeneration that may develop into knee osteoarthritis, all of which seriously affect the life quality of patients [5, 6]. Injured or torn meniscus has a limited self-healing capacity as only the periphery regions of meniscus has the blood supply. Some meniscus tears can be surgically repaired, but treatments for central tears or large injuries are very limited. The well-established treatment option is meniscal allograft transplant, i.e., to replace the damaged meniscus with a meniscus from a cadaver donor, but the clinical outcome is not satisfactory [7, 8]. The main problems include the limited availability of materials, incomplete functional recovery, and tissue rejection [9–12]. Meanwhile, due to the large individual differences in the geometry of the meniscus among the population, the geometry mismatch of the meniscus between the donor and the recipient greatly hinders the efficacy of the replacement therapy. Therefore, development of customized biocompatible alternatives to recapitulate native tissue complexity is of great clinical significance for meniscus engineering.

Many types of biomaterials have been explored to create meniscus scaffolds for meniscus regeneration. The clinical application of collagen I-based scaffold CMI^®^ (Stryker Corporation) and polymer-based scaffold Actifit^®^ (Orteq Sports Medicine), have demonstrated the feasibility of utilizing biocompatible scaffolds for treatment of meniscus injuries [13–16]. However, these acellular scaffolds have the problems of insufficient mechanical support or/and poor postoperative functional recovery [17–20]. The strategy that utilizes a 3D scaffold as a carrier to deliver mesenchymal stem cells (MSCs) to the defect area has received a lot of attention and demonstrates the efficacy in tissue regeneration [21–24]. A number of natural and synthetic materials coupled with MSCs have been investigated and applied to promote chondrogenic differentiation and cartilage regeneration, such as natural components [25–27], graphene/graphene oxide [28, 29], synthetic polymers [29–31], or hybrid materials [31, 32].

An ideal implantable scaffold should mimic the complexity of patients’ native meniscus by possessing comprehensive characteristics, including precision geometry, appropriate mechanical properties, tissue-regeneration bioactivity and practical logistics. 3D printing of synthetic polymer scaffolds have held several advantages including ample supply, easy manipulation and the potential to achieve appropriate geometry and mechanical properties under controlled fabrication methods. Meanwhile, scaffolds designed in the porous structure were proved to support cartilage tissue formation through interconnected pores by providing enhanced nutrient transportation and cell infiltration as well as increased surface for cell attachment and cell proliferation [33, 34]. The key factors that determine porous scaffolds’ mechanical properties include the choice of biomaterials, pore size, porosity and interconnectivity. A number of synthetic polymers have been studied for multiple origin tissue engineering [2]. Tailored scaffolds with precision geometry and controlled pore size and shape can be achieved by computer aided design and rapidly developed 3D printing technology.

Surface properties of 3D porous polymer scaffold play a critical role in tissue-regeneration bioactivity. It is well-known that some ECM play a guiding role in cell functions through its interaction with cell surface receptors such as integrin, including cell adhesion, morphology, proliferation, migration and differentiation [35, 36]. Coating ECM components has been widely used in cell type-specific tissue culture, and coating them on 3D polymer scaffold enhances material wettability and cell adhesion epitopes and thus favors cellular function and tissue regeneration [37, 38]. Among them, collagen I and fibronectin are two widely used ECM components for scaffold surface modification [37–40].

In the present study, we generated porous PU meniscus scaffolds through 3D cryo-printing technology, with tailored geometry and adjusted porosity in order to better replicate native meniscus. Mechanical property, scaffold morphology and biocompatibility along with other material characterizations of the 3D porous PU scaffold was assessed for its feasibility in tissue engineering. PU scaffolds were coated with collagen I and fibronectin through physical absorption to explore the optimal surface treatment for cell adhesion and growth. In situ cell proliferation and chondrogenic differentiation on the PU scaffold was extensively evaluated using hMSCs. Our results demonstrates the fabricated biocompatible porous meniscus-like scaffolds greatly promote hMSCs growth and chondrogenic differentiation, and are suitable for cartilage regeneration and meniscus tissue engineering.

## Materials and methods

### Preparation of 3D meniscus scaffolds

Based on the physiological anatomical structure and geometry of human meniscus, porous and mesh meniscus models with ranging pore diameter from 0.25 mm to 0.7 mm (porosity 20–60%) to reflect different mechanical strength was designed. The designed 3D model was printed using the patent-owned cryo-printing method (China patent # CN 209966665 U). Dissolved PU material was printed in the fashion of layer by layer below − 20 °C, followed by vacuum freeze-drying process at − 70 °C and the final surface activation coating process under low vacuum condition.

### Compressive mechanical testing

To study the mechanical properties of PU scaffolds with different pore sizes, the scaffolds (5 mm × 5 mm × 5 mm) were mechanically evaluated under compressive testing using an ZwickRoell A624809 (ZwickRoell, Ulm, Germany). 3 samples of each porosity were prepared for the compressive test. A compression rate of 0.5 mm/s was used following the ASTM standards. Results were analyzed using the TestXpert V10.11 software. The compressive modulus was determined by calculating the slope of the initial linear region of the stress–strain curve.

### 3D porous PU scaffold morphology

Before imaging, scaffold samples (~ 5 mm × 5 mm × 2 mm) were cut and placed onto a metallic stub by a double-sided carbon tape and coated with platinum using a Hitachi MC1000 Ion Sputter Coater for about 60 s. The morphology of 3D porous PU scaffold was observed by a Hitachi UHR FE-SEM SU8010 (Tokyo, Japan) at an acceleration voltage of 3 kV.

### Other characterizations of prepared PU scaffolds

1. Fourier Transform Infrared Spectroscopy (FT-IR). Infrared ATR spectra of PU scaffolds were measured with a Nicolet IS5 spectrometer (Thermo Scientific, USA) from 4000 to 400 cm^−1^ after 32 scans of each sample. The absorbance spectrum was measured with a wavelength resolution of 4.0 cm^−1^.
2. X-ray Powder Diffraction (XRD). Experiments were performed on the scaffold samples with Bruker D8 Advance X-ray diffractometer (Bruker, Germany) using a Cu Kα source in the 2θ angles range between 5° and 90° with a scan rate of 3°/min.
3. X-ray Photoelectron Spectroscopy (XPS). XPS measurements were obtained with an Escalab 250Xi spectrometer (Thermo Scientific, USA). For survey spectra, the pass energy was set to 100 eV (with scanning step 1 eV). For specific spectra including C, O, N and S element scan, the pass energy was set to 20 eV (with scanning step 0.05 eV). The samples of PU and FN-treated PU (PU/FN) scaffolds for cell culture were tested as-prepared.
4. Contact Angle. The contact angle measurements of PU and PU/FN scaffolds were performed using a Theta Flex optical tensiometer (Biolin Scientific, Sweden) with subsequent evaluation of the contact angle from the images using the See Software 7.0. During the measurement, 2 µL of deionized water were dropped on the test material, and each sample was measured five times and averaged.
5. Zeta Potential. The zeta potentials of PU and PU/FN scaffolds were analyzed using a SurPASS 3 Electrokinetic analyzer (Anton Paar, Austria) in the pH range between 3 and 10. All measurements were performed at 25 °C and repeated three times.
6. Thermogravimetric Analysis (TGA/DTG). TGA/DTG analysis was carried out using the TGA2 instrument (Mettler Toledo, Swiss) with 5 mg mass sample for each scaffold. The experiment was carried out under N_2_ atmosphere from 25 °C to 800 °C at the heating rate of 10 °C/min.

### Biocompatibility test

Adult male SD rats (250–300 g) were obtained from Experimental Animal Institute of Medical College of Zhejiang University. The rats were housed in a controlled environment under standard conditions of temperature and humidity and an alternating 12-h light and dark cycle. After anesthesia, the back skin of the rat was opened to expose the subcutaneous fascia and subcutaneous tissue. The scaffolds (~ 5 mm × 5 mm × 2 mm) were respectively implanted into the fascia of the rat back skin, and then the subcutaneous fascia and skin are sutured in layers. Four weeks later, the rats were sacrificed, and the skin and subcutaneous tissues of the original surgical site were opened to observe the local inflammation and the healing status of the subcutaneous tissues. Explants were fixed in 4% paraformaldehyde for 48 h, and then dehydrated in series ethanol before embedding in paraffin. 5-μm-thick sections were used for hematoxylin and eosin (HE) staining.

### Cell culture and seeding onto meniscus scaffolds

Human umbilical cord-derived mesenchymal stem cells (hMSCs) and complete stem cell growth medium were obtained from Stem Biotechnology Co., Ltd. (HangZhou, China). hMSCs were cultured in complete growth medium supplemented with 1 × Antibiotic–Antimycotic (#15240-062, Thermo Fisher, USA) in T-75 flasks (#3290, Corning, USA) at 37 °C, 5% CO_2_, and 95% relative humidity. When cells reach 80% confluence, cells were detached by TrypLE Express (#12604021, Thermo Fisher, USA) and plated in a new flask at 1:5 density, or seeded onto the scaffold.

The scaffolds are subjected for sterilization and ECM coating treatment prior to cell seeding. First, the scaffolds were soaked in 75% ethanol for 1 h, then placed in a biological safety cabinet for 48 h to remove residual ethanol and sterilized under UV light for 1 h on each side. To promote cell adhesion and growth, the scaffolds were pre-treated with fibronectin (50 µg/mL or 200 µg/mL, #MX0926, MKbio, China) or collagen I (1 mg/mL, #5162, Advanced BioMatrix, USA) for 48 h at 4 °C. Afterwards, the scaffolds were rinsed once with phosphate buffered saline (PBS, #CR-20012, Cienry, China) and placed in a 24-well plate (#3524, Costar, USA). For cell seeding, 1 mL cells in complete growth media at the density of 2 × 10^5^ cells/mL was placed on the top center of the scaffolds. After 24 h, the scaffold was transferred to a new well for further culture.

### Cell proliferation and viability

The proliferation of hMSCs were assayed using CellTiter-Glo luminescent cell viability assay (CTG, #G7572, Promega, USA), according to manufacturer’s instructions. Briefly, after hMSCs were cultured on the scaffolds for 3, 7 and 14 days, the scaffold was transferred into an empty well containing 200 μL medium. Subsequently, 100 μL CTG detection reagent was added to the well, and incubated for 20 min in the dark. Luminescence signal was quantitated using Tecan Spark microplate reader (Tecan Group Ltd., Swiss). The luminescence signal is proportional to the number of live cells, so it was used to monitor the hMSCs proliferation on the scaffold.

Live/dead assay was also performed to assess cell viability and cell distribution on the scaffold. Calcein-AM dye (#C131116, Aladdin, China) and propidium iodide (PI, #P266304, Aladdin, China) were used to label live cells and dead cells, respectively. Hoechst dye (#B1123845, Aladdin, China) was used to stain cell nuclei. The staining process was as follows: A staining solution containing 2 μM calcein-AM, 4.5 μM propidium iodide and 5 μg/mL hoechst were prepared in PBS. The scaffold was thoroughly rinsed with PBS and was incubated with 500 µL of staining solution for 30 min. The staining solution was discarded and the scaffold was washed with PBS before imaging through a laser confocal microscope (Olympus FV3000, Japan) or fluorescent microscope (Olympus IX73, Japan).

### Chondrogenic differentiation

Chondrogenic differentiation media consisted of hMSCs growth media supplemented with 10 ng/mL TGF-β1 (#CA59, Novoprotein, China), 100 nM dexamethasome (#HY-14648, MedChemExpress, USA), 40 μg/mL L-proline (#P5607, Sigma, USA), 50 μg/mL ascorbate-2-phosphate (#49752, Sigma, USA) and 1% ITS (#I2521, Sigma, USA) [41–43]. When hMSCs on the scaffolds cultured in growth media reached high density, the scaffolds were separated into two groups: (1) Chondrogenesis culture: the scaffolds were cultured in chondrogenesis media; (2) Growth culture: the scaffolds were cultured in growth media. The culture medium was renewed every 3 days, and the scaffolds were retrieved for testing at 14 days and 21 days.

### Immunofluorescence staining

At the end of the chondrogenesis or growth culture, the final tissue constructs were washed with PBS and fixed with 95% v/v ice ethanol at 4 °C for 20 min. Afterwards, samples were permeabilized with 0.3% v/v Triton X-100 at room temperature for 20 min and washed three times (5 min each time) with PBS. Then the samples were blocked in 3% bovine serum albumin (BSA, #ST023, Beyotime, China) solution for 1.5 h at room temperature. The tissue constructs were then incubated with primary antibody for collagen II (1:800 v/v, mouse collagen II monoclonal antibody, #ab34712, Abcam, UK) and ACAN (1:400 v/v, rabbit ACAN monoclonal antibody, #MA3-16888, Invitrogen, USA) for 2 h at room temperature. The samples were washed three times with 0.2% v/v Tween-20 in PBS and incubated with goat anti-mouse IgG secondary antibody (1:500 v/v, #ab150115, Abcam, UK) and goat anti-rabbit IgG secondary antibody (1:500 v/v, #ab150080, Abcam, UK) in the dark for 2 h at room temperature. Hoechst fluorescent dye (1.5 µg/mL in PBS, #B1123845, Aladdin, China) was used to counterstain nuclei. The samples were then washed five times with 0.2% v/v Tween-20 in PBS, and imaged using laser confocal microscope (Olympus FV3000, Japan).

### Western blot

Samples were lysed in ice RIPA lysis buffer (#P0013B, Beyotime, China), incubated on ice for 30 min, and centrifuged at 12,000*g* for 10 min. The protein concentration was determined with a bicinchoninic acid (BCA) protein assay (#P0010, Beyotime, China). Equal amounts of protein extracts were fractionated by 12% sulfate–polyacrylamide gel electrophoresis (SDS-PAGE) and transferred onto polyvinylidene fluoride (PVDF) membranes. Membranes were incubated with the following primary antibodies overnight at 4 °C: collagen II (mouse collagen II monoclonal antibody, #ab34712, Abcam, UK) and aggrecan (rabbit ACAN monoclonal antibody, #MA3-16888, Invitrogen, USA), ERK (rabbit monoclonal ERK antibody, #4695, Cell Signaling Technology, USA) and pERK (rabbit monoclonal pERK antibody, #9101, Cell Signaling Technology, USA). GAPDH was used as an internal loading control and detected with a mouse monoclonal antibody (#ab8245, Abcam, UK). The secondary HRP-conjugated antibody (goat anti-rabbit antibody, #BK-R050; goat anti-mouse #BK-M050, Bioker, China) was used for detection. The antibody associated protein bands were revealed using the Ncm ECL Ultra Western blotting kit (#P10100, Ncm Biotech, China), and visualized using the Touch Imager XLi (e-BLOT, China).

### Quantitative real-time PCR analysis

The mRNA expression of stem cell and cartilage-specific genes were analyzed from corresponding samples. Total RNA was isolated using the TRIzol reagent (#9109, Takara, Japan), and were reverse transcribed into first-strand cDNA using Hiscript III RT SuperMix Kit (#R323-01, Vazyme, China) following the manufacturer’s guideline. PCR amplification was performed using the ChamQ Universal SYBR qPCR Master Mix (#Q711-02/03, Vazyme, China) on Real Time PCR Detection System (BioRad Laboratories, USA) according to the manufacturer’s recommendations. The reactions were carried out in triplicate and conditions were as follows: 95 °C for 30 s, 40 cycles of 95 °C for 10 s and 60 °C for 30 s, followed by a melting curve analysis at 95 °C for 15 s, 60 °C for 60 s and 95 °C for 15 s. The target gene expression was normalized against the housekeeping gene GAPDH under the same conditions, and the relative fold difference of expression levels was calculated using the 2^−ΔΔCt^ algorithm. The primer sequences used for amplification are specified in Table 1.

**Table 1:** Primer sequences used for quantitative real-time PCR analysis of stem cell and chondrogenesis-associated specific genes

Target gene	Sequence
GAPDH	F: TGTTGCCATCAATGACCCCTT
	R: CTCCACGACGTACTCAGCG
ACAN	F: TGAGGAGGGCTGGAACAAGTACC
	R: GGAGGTGGTAATTGCAGGGAACA
SOX9	F: TTCATGAAGATGACCGACGA
	R: CACACCATGAAGGCGTTCAT
COL2A1	F: GGCAATAGCAGGTTCACGTACA
	R: CGATAACAGTCTTGCCCCACTTA
COL1A1	F: TCTGCGACAACGGCAAGGTG
	R: GACGCCGGTGGTTTCTTGGT
COL10A1	F: CCAGGTCTGGATGGTCCTA
	R: GTCCTCCAACTCCAGGATCA
CD45	F: ACGAAGCTCTTAGCGTCAGG
	R: CTCTCGGGTGGAGTCTTCTG
CD73	F: CAGTACCAGGGCACTATCTGG
	R: AGTGGCCCCTTTGCTTTAAT
CD90	F: GACAGCCTGAGAGGGTCTTG
	R: CCCAGTGAAGATGCAGGTTT
CD105	F: CACTAGCCAGGTCTCGAAGG
	R: CTGAGGACCAGAAGCACCTC

### Statistical analysis

All data was plotted as mean ± standard deviation. Unless stated otherwise, three experimental replicates (n = 3) were performed. Statistical significance was determined by a two-tailed Student t-test and one-way ANOVA with post hoc Tukey test using GraphPad Prism, and statistically different values were considered for p-value < 0.05 (*p < 0.05, **p < 0.01, and ***p < 0.001).

## Results

**Fig. 1:**
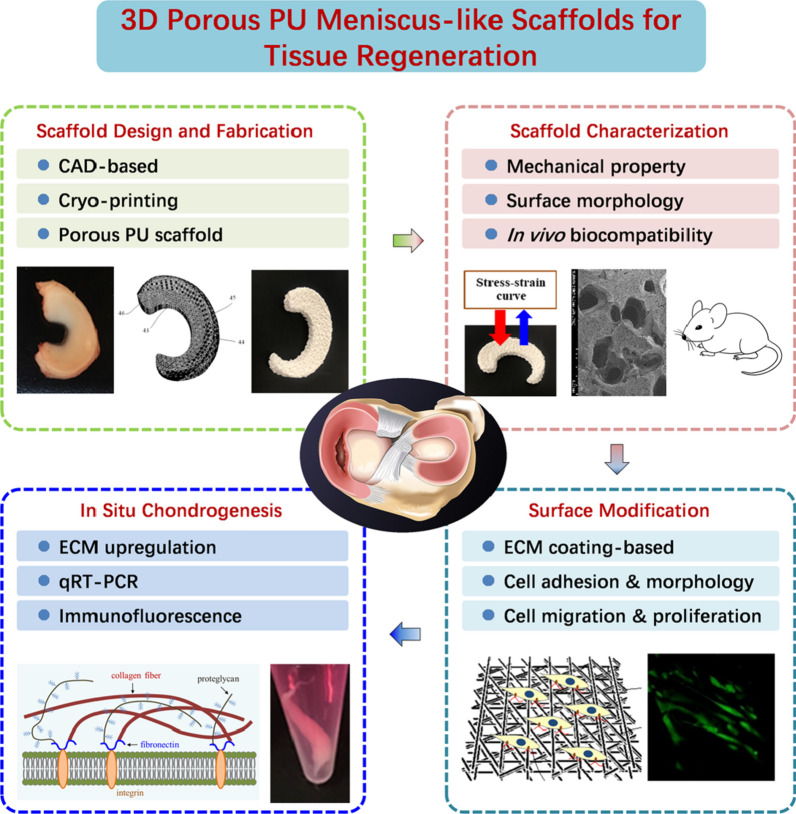
Schematic overview of the study of 3D porous PU meniscus constructs for tissue regeneration

The objectives of this study were to (1) design and fabricate meniscus scaffolds via cryo-printing; (2) characterize the mechanical properties and in vivo biocompatibility of the constructs through subcutaneous implantation; (3) investigate the effects of scaffold surface modification on hMSCs function, focusing on cell adhesion, migration, proliferation and long-term viability; (4) evaluate in situ chondrogenesis of hMSCs for potential meniscus regeneration (Fig. 1).

### Fabrication and characterization of 3D porous meniscus scaffolds

**Fig. 2:**
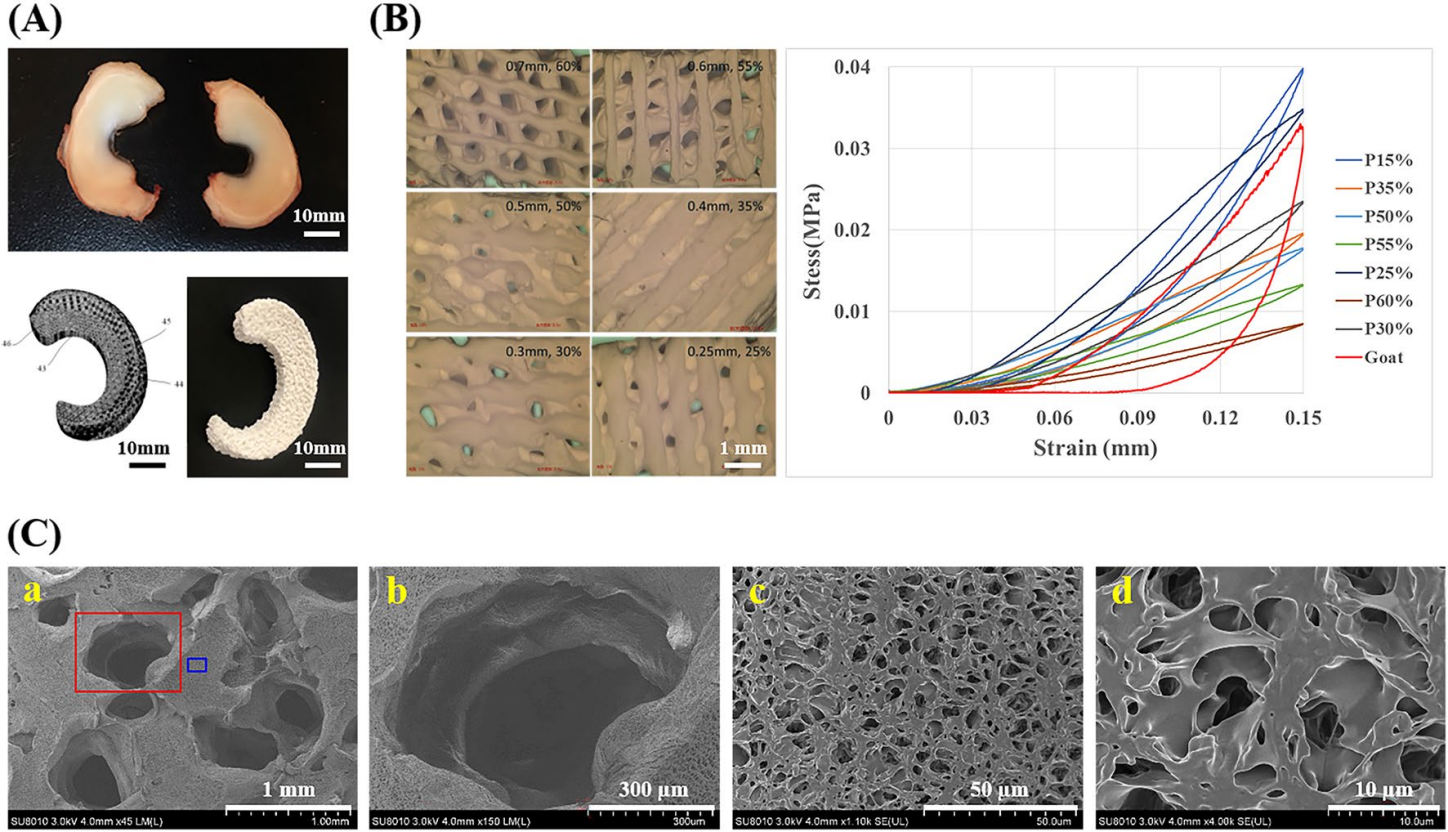
3D printed meniscus-like scaffold characterization: **A** Goat meniscus and designed model; **B** Scaffold optimization in porosity; **C** Representative SEM images of 3D printed scaffold. Scale bar **a** 1.0 mm; **b** 300 µm; **c** 50 µm; **d** 10 µm

To mimic the anatomical structure and mechanical properties of native meniscus, 3D porous meniscus constructs were designed (Fig. 2A), which are expected to provide sufficient space for cell growth and cartilage formation while matching the geometry of native meniscus and its mechanical support along with its degradation process.

A patented cryo-printing method was employed to generate the meniscus-mimicking scaffolds by layers using polyurethane-based material. Varied pore size was achieved by a shift of distance and angle between layers. When the pore diameter was adjusted from 0.15 mm to 0.7 mm, the porosity of scaffolds varied from 15 to 60%, and thus reflect different mechanical strength of the scaffolds. Compressive mechanical tests showed that scaffolds with higher porosity displayed lower mechanical strength, based on the calculated compressive modulus value obtained from the stress–strain curve in Fig. 2B. The mechanical property of scaffolds with 25% porosity (0.25 mm diameter in average) was very close to that of the goat meniscus, which has been reported to be close to human meniscus [2, 44].

The morphology of 3D PU scaffold with 25% porosity was observed by SEM. As shown in Fig. 2C, the scaffolds presented with the obvious irregular macropores structure with diameters ranging from 200 µm to 500 µm, and the dense micropores structure with diameters ranging from 1 µm to 10 µm. Those irregular pores provide the space for nutrition transportation, cell adhesion, migration and tissue formation.

**Fig. 3:**
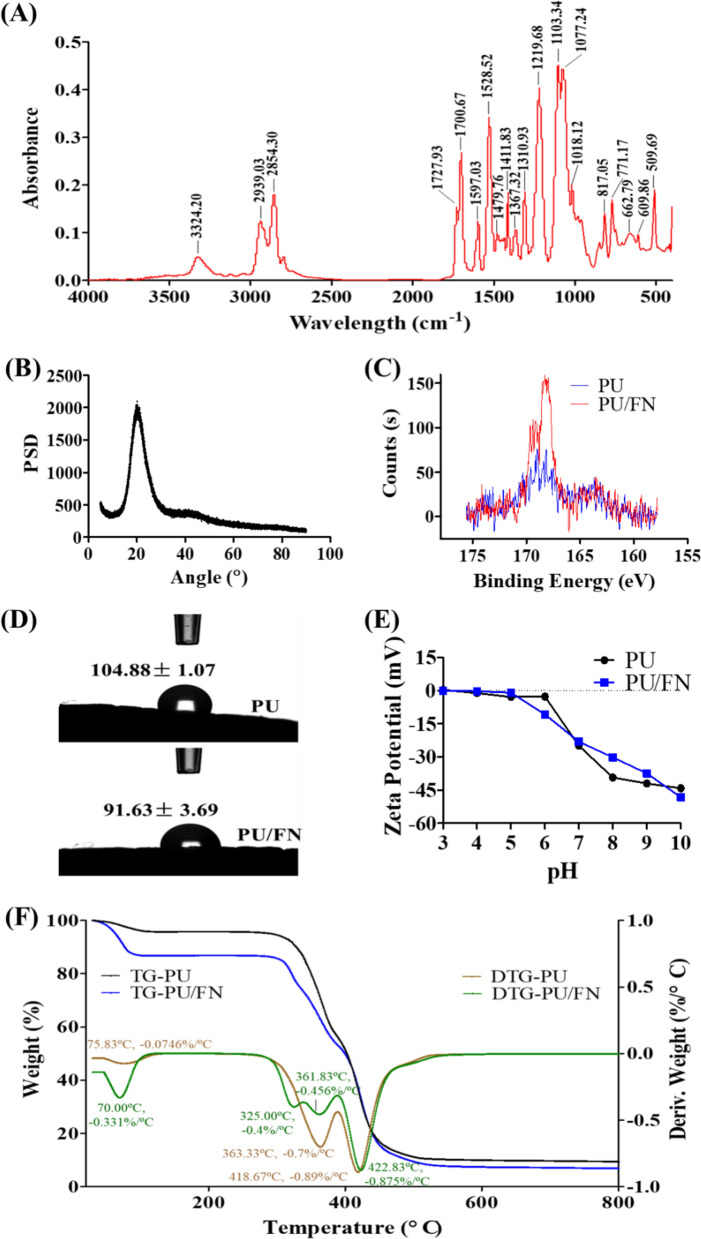
3D printed PU scaffold characterizations: **A** FT-IR spectra of PU scaffolds; **B** X-ray diffraction analysis of PU scaffolds; **C** X-ray photoelectron spectroscopy of S2p scan for PU and PU/FN scaffolds confirming FN absorption on the scaffolds; **D** Contact angles of water on PU and PU/FN scaffolds; **E** Zeta potentials of PU and PU/FN scaffolds in the pH range from 3 to 10; **F** TG/DTG curves of PU and PU/FN scaffolds under N_2_ atmosphere

The PU scaffolds and FN-treated PU scaffolds were also characterized by the following analysis: FT-IR, XRD, XPS, contact angle, zeta potential and TGA/DTG. FT-IR analysis of PU scaffolds was shown in Fig. 3A. The characteristic absorptions peaks of the PU were observed at 3324 cm^−1^ (N–H stretching frequency), 2939 and 2854 cm^−1^ (–CH_2_– and –CH_3_ stretching frequencies), 1728 and 1701 cm^−1^ (carbonyl urethane stretching), 1529 cm^−1^ (CHN N–H + C–N vibration), 1220 cm^−1^ (coupled C–N and C–O stretching), and 1103 and 1077 cm^−1^ (C–O stretching). Band features of the pre-polymer isocyanates at ~ 2312 cm^−1^ were not observed. FN-coated PU scaffolds exhibited the same spectra profile as neat PU scaffolds (data not shown). Figure 3B showed the X-ray diffraction patterns of PU scaffolds. The diffraction peak appeared at 2θ angles around 20.9°, no extra peaks were observed. X-ray photoelectron spectroscopy was employed to analyze the elemental composition of the PU scaffolds and FN-treated scaffolds. Specific scan for C, O, N and S element was performed besides XPS survey (Survey spectra was not shown). Compared to PU scaffolds, enhanced S2p signal was observed in FN/PU scaffolds as shown in Fig. 3C.

The hydrophilicity and surface charge of the prepared scaffolds were evaluated by the contact angle and zeta potential detection. As shown in Fig. 3D, the determined contact angle of water was 104.88 ± 1.07 for PU scaffolds, whereas it was 91.63 ± 3.69 for PU/FN scaffolds. The surface charge of the prepared scaffolds was studied by zeta potential analysis. It is graphically presented in Fig. 3E as a function of pH. In the case of the bare PU scaffolds, the zeta potential was positive (0.42 mV) at pH 3, became negative (− 1.15 mV) at pH 4 and kept decreasing to -44.13 mV at pH 10. Similar to the PU scaffolds, the zeta potential for the FN/PU scaffolds was 0.03 mV at pH 3, became negative (− 0.25 mV) at pH 4 and decreased to − 48.25 mV at pH 10. The water content and thermal stabilities of prepared scaffolds were analyzed by TGA/DTG. The TG curve for PU scaffolds presented in Fig. 3F showed two steps of thermal decomposition at high temperatures: (1) 22% of mass loss between 300 °C and 360 °C; (2) 62% of mass loss occurs between 360 °C and 525 °C. A small % weight loss observed in the temperature range 45–120 °C is apparently associated with adsorbed water. As for the FN-soaked PU scaffolds, it also presented the two similar steps of thermal decomposition, but with more % weight loss of water (13.3% vs. 4.3%).

**Fig. 4:**
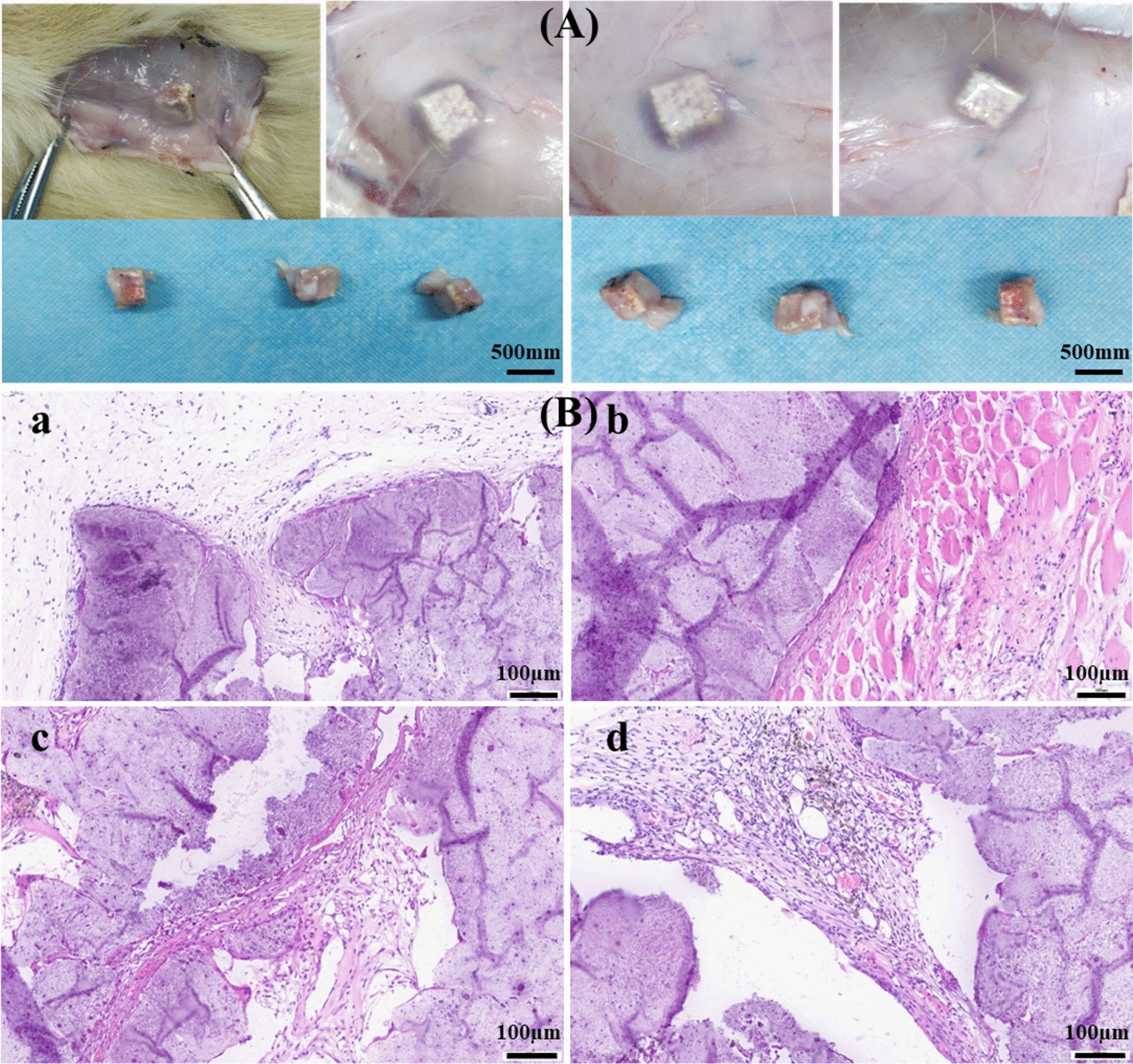
Biocompatibility of scaffolds evaluated by subcutaneous implantation on SD rats followed by HE staining (n = 3). **A** Observation of the skin and subcutaneous tissues of the original surgical site in the fascia of the rat back skin. **B** Representative H&E staining images of the tissues surrounding the implants (**a** and **c**), and interior tissues of the explants (**b** and **d**)

The in vivo biocompatibility of 3D porous meniscus scaffolds were evaluated by subcutaneous implantation in SD rats followed by HE staining. Observed from the rat subcutaneous tissue models in Fig. 4A, there was no obvious local inflammation, no redness, swelling, and purulent. The scaffolds were embedded in the subcutaneous fascia tissue, and the fusion with the tissue grew very well. The incision and skin were well healed. The rats were healthy and active, indicating that the scaffolds had good biocompatibility in vivo. Histologically, no fibrous encapsulation was observed surrounding the scaffold (Fig. 4B), implying surface modification using our approach significantly improved its biocompatibility. Moreover, new tissue grown into the interior structure of the scaffolds with the presence of capillary vessels, indicating the scaffold could effectively induce tissue ingrowth and local vascularization, which are both considered critical in the context of meniscus regeneration.

### Cell adhesion and proliferation on 3D porous meniscus scaffolds

**Fig. 5:**
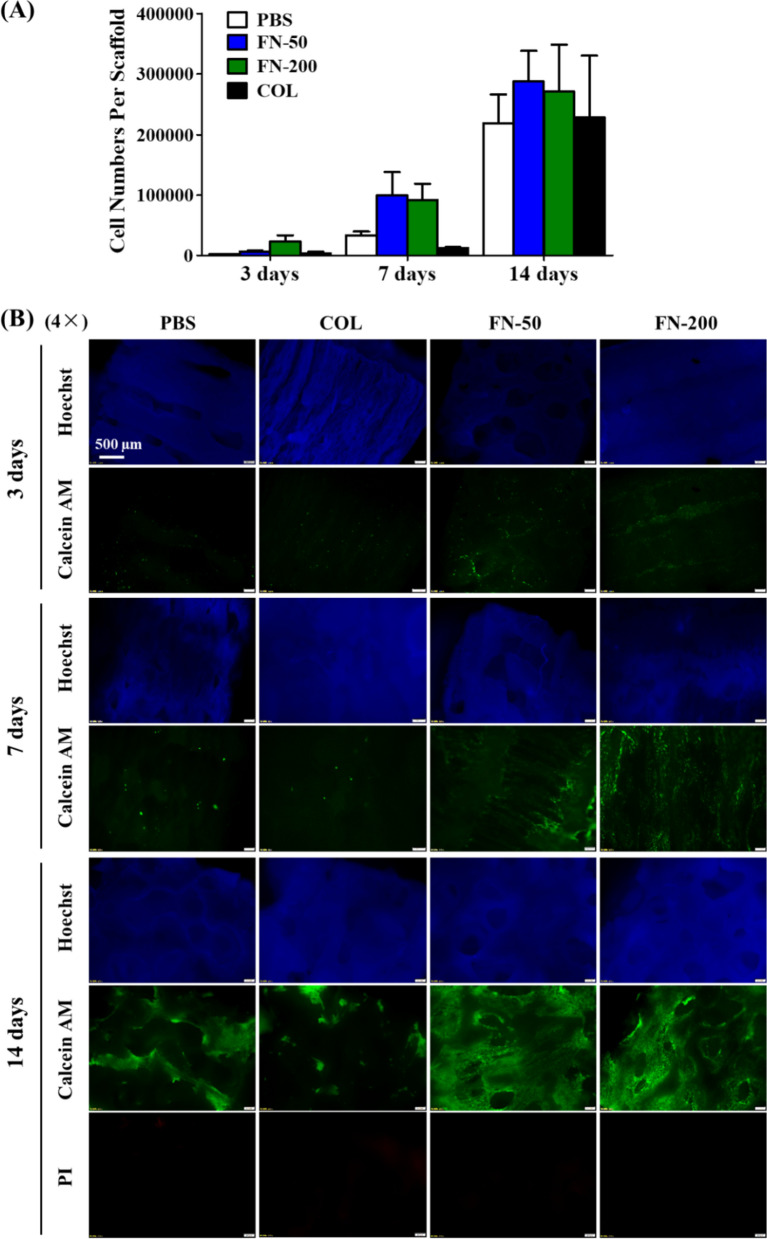
hMSC cell growth over time on treated scaffolds (PBS: no protein treatment; COL: 1 mg/mL collagen I; FN-50: 50 µg/mL fibronectin; FN-200: 200 µg/mL fibronectin) assessed by **A** cell viability assay (n = 3) and **B** live/dead staining

To facilitate cell adhesion and proliferation, the fabricated porous PU scaffolds were pretreated with FN and collagen I (COL) and hMSC cell proliferation on scaffolds was monitored in two weeks by cell viability assay and live/dead staining. Both Fig. 5A and B showed that hMSCs numbers clearly increased from 3 to 14 days on either group of scaffolds. More cells were attached on FN-treated scaffolds than COL-treated scaffolds at 3 days and 7 days post seeding, whereas cell numbers on COL-treated scaffolds was similar to that on PBS-treated scaffolds. No dead cells were observed in cultures of 14 days on the porous scaffolds with either treatment (Fig. 5B) even in the interior of the scaffold (data not shown).

**Fig. 6:**
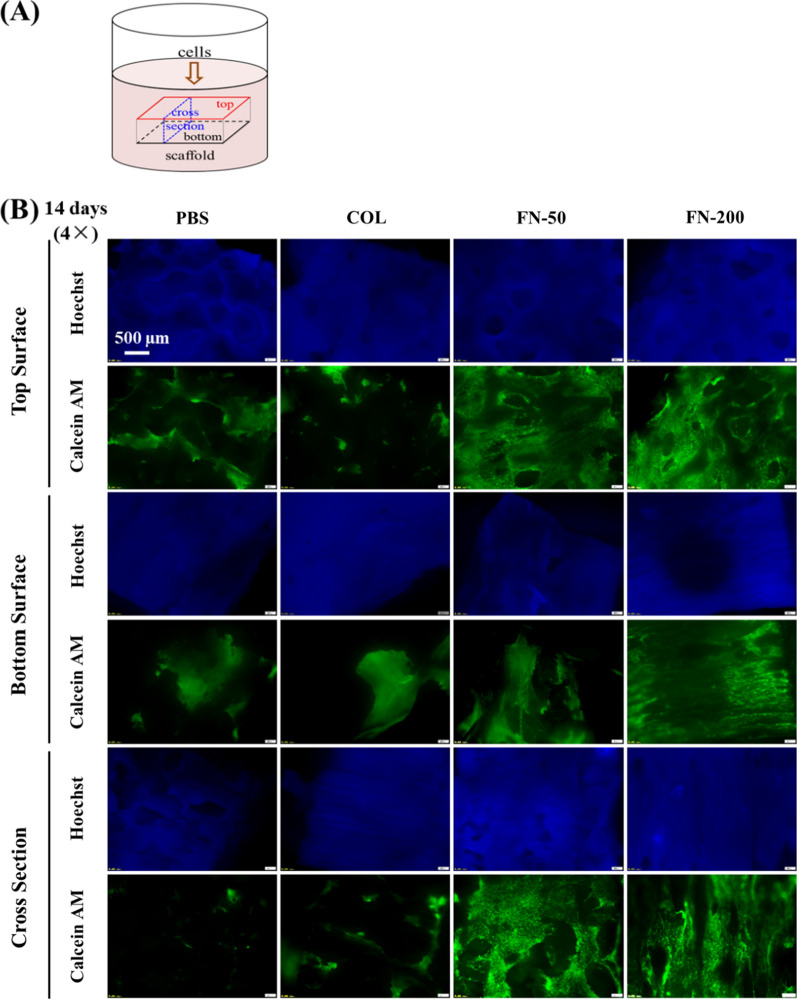
**A** Scheme diagram of cell seeding and different sections of a scaffold; **B** hMSCs distribution on scaffolds under different treatments

**Fig. 7:**
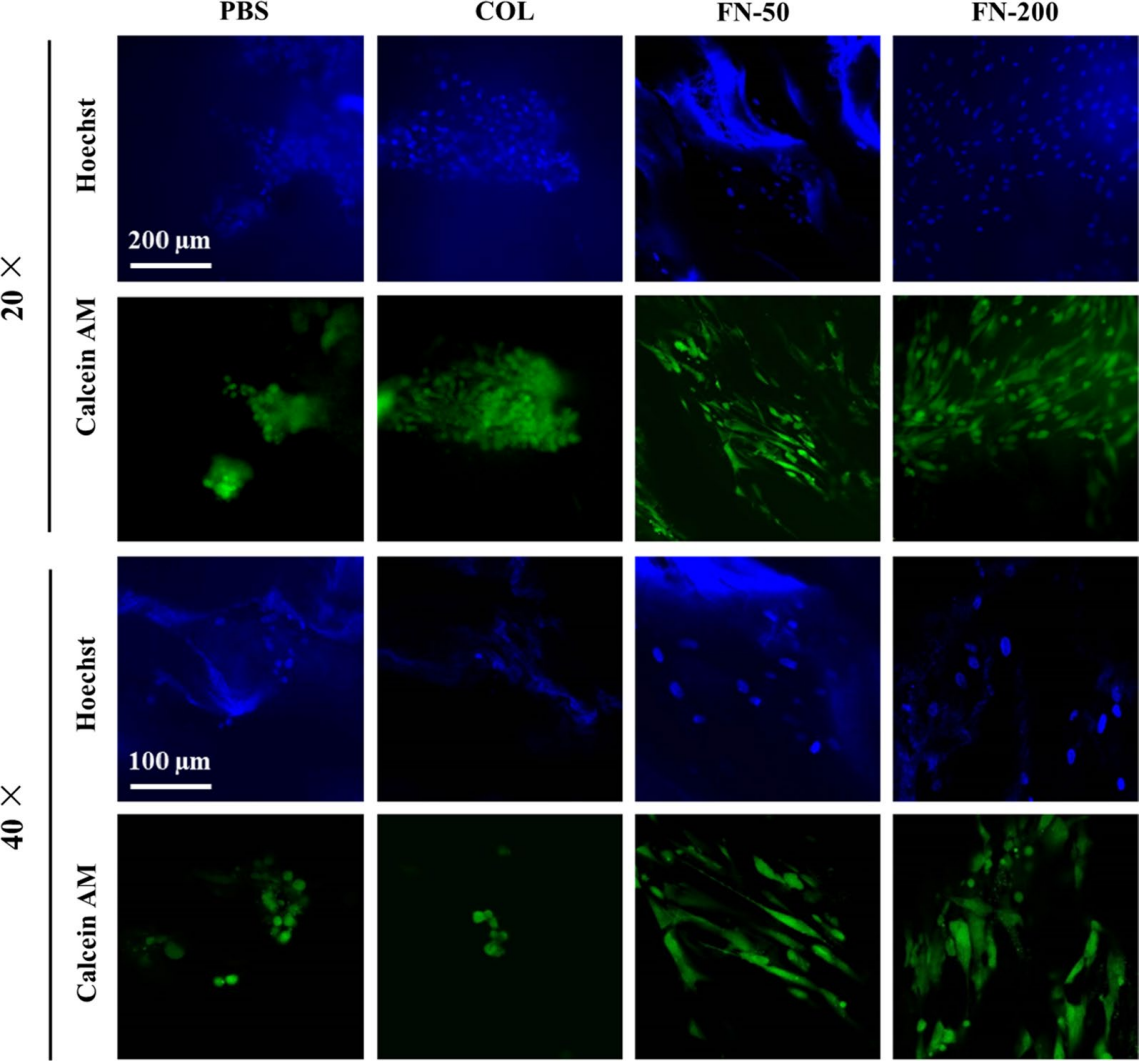
hMSCs morphology on scaffolds under different treatments at higher magnification

To further evaluate cell distribution and migration in scaffolds, live/dead staining of 14-day cultures in Fig. 6 showed that hMSCs were observed on both top and bottom surfaces, but more cells were present on the top surface, which was attributed to the cells precipitation on the top surface after cell seeding. More importantly, hMSCs were observed in the interior of the scaffolds under high magnification, which was obvious in FN-treated scaffolds (Fig. 6B). Although similar number of cells grown on materials treated with either FN, COL or PBS after 14-day incubation, hMSCs maintained its original cell morphology in a spindle shape and distributed more uniformly on FN-treated scaffolds, whereas cells on COL or PBS-treated materials presented in an oval shape, and were unevenly distributed in clusters. The difference in cell morphology due to surface modification can be clearly observed from high magnification images in Fig. 7.

### Chondrogenic differentiation on 3D porous meniscus scaffolds

**Fig. 8:**
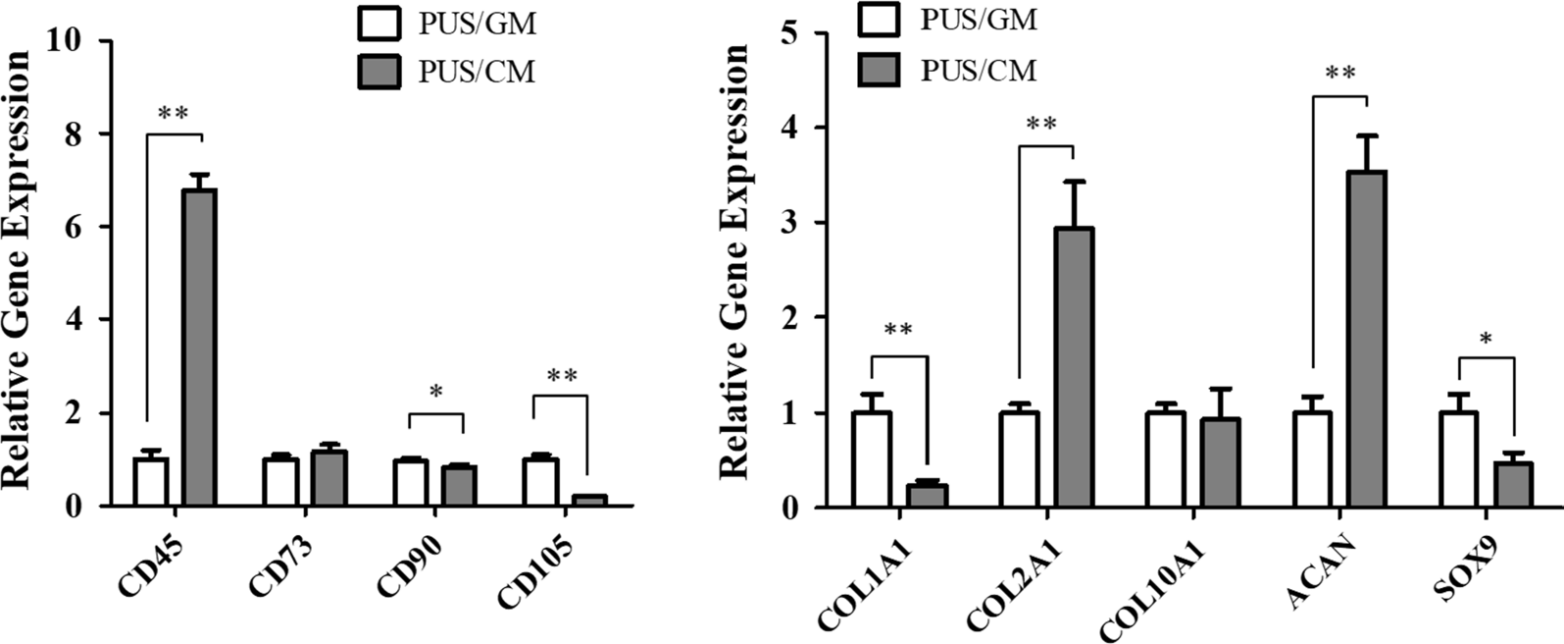
Chondrogenic differentiation of hMSCs cultured for 14 days on scaffolds assessed by qRT- PCR analysis of relative gene expression levels (n = 3). GAPDH was used as an internal control, and the values are given as the fold change compared to PUS/GM group. *p < 0.05 and **p < 0.01

To explore the chondrogenic differentiation of hMSCs on artificial meniscus scaffold, the relative gene and protein expression level of chondrogenic specific markers were determined by qRT-PCR and immunostaining. Human umbilical cord-derived mesenchymal stem cells are characterized for positive expression of CD90, CD105, CD73, and negative expression of CD45 [45]. Therefore, these four stem cell-associated genes were also included in qRT-PCR analysis. Four different culture protocols were employed to compare the gene expression profiles of hMSCs under growth and chondrogenesis condition, namely (a) TC flask/ growth media (TCF/GM), (b) TC flask/chondrogenesis media (TCF/CM), (c) PU scaffold/growth media (PUS/GM), (d) PU scaffold/chondrogenesis media (PUS/CM). Figure 8 showed the gene expression levels of chondrogenesis-specific markers and stem cell-associated markers after 14 days of hMSCs culture on scaffolds, i.e., under PUS/GM and PUS/CM culture protocols. Only two essential chondrogenic biomarkers ACAN and COL2A1 showed 2.9-fold and 3.5-fold increased expression on scaffolds with 14 days of TGF-β1 induction. In contrast, the expression levels of COL1A1 and SOX9 decreased by 4.3-fold and 2.1-fold. Under differentiation culture, CD45 expression increased by 6.8-fold and CD105 expression decreased by fivefold.

**Fig. 9:**
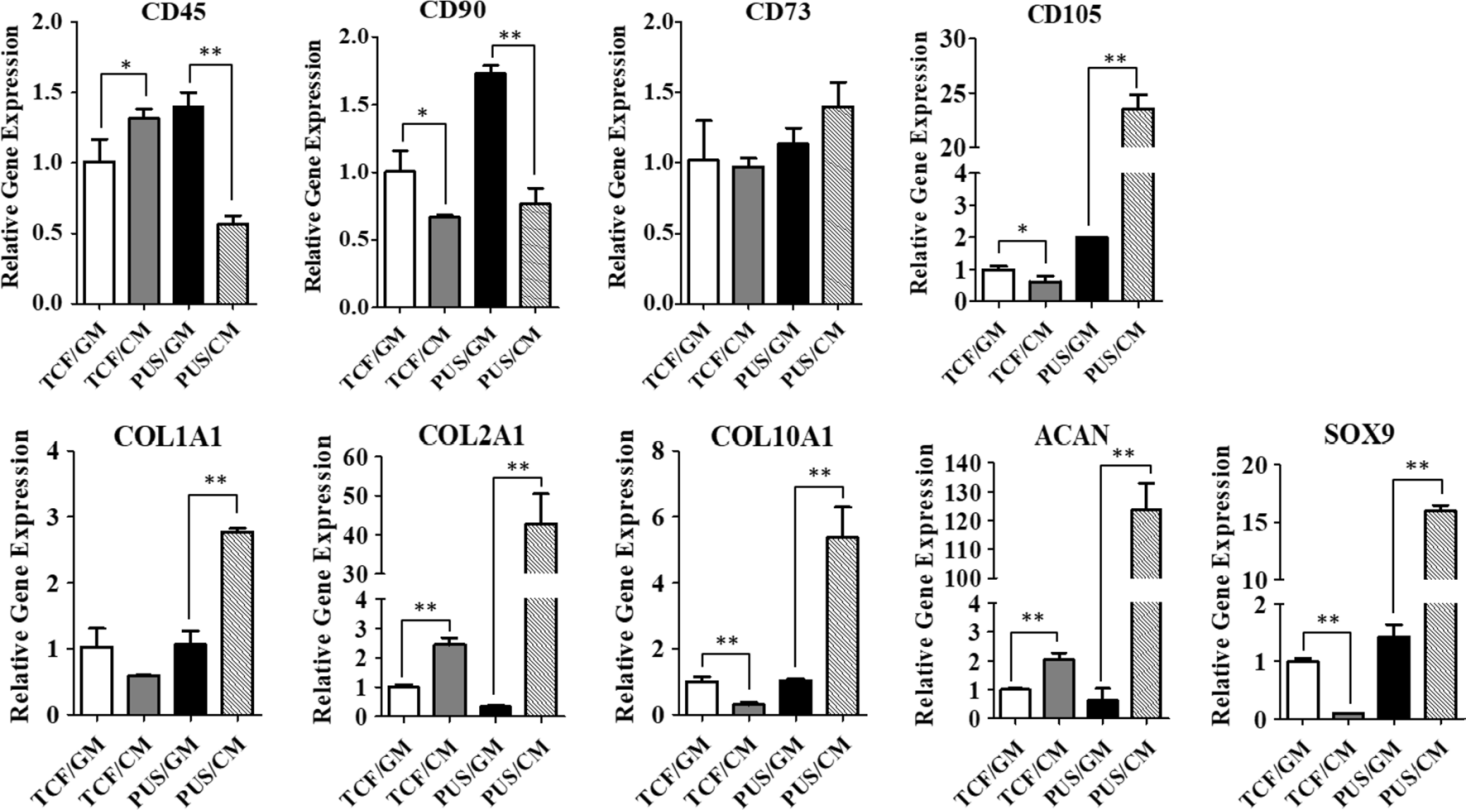
Chondrogenic differentiation of hMSCs cultured for 21 days on scaffolds assessed by qRT-PCR analysis of relative gene expression levels (n = 3). GAPDH was used as an internal control, and the values are given as the fold change compared to TCF/GM group. *p < 0.05 and **p < 0.01

Figure 9 demonstrated expression levels of these genes after 21 days of hMSCs culture under four protocols. As for gene expression of stem cell-associated markers, overall no significant difference between four culture groups was observed, except for CD105 with 11.8-fold increase, and CD45 and CD90 with 2.5-fold and 2.3-fold decrease in PUS/CM group compared to PUS/GM group. Compared with PUS/GM protocol, PUS/CM protocol significantly enhanced the gene expression of ACAN and COL2A1, with the upregulation as high as 195-fold and 129-fold, respectively. Moreover, other chondrogenesis specific genes including COL1A1, COL10A1 and SOX9 were also significantly upregulated on scaffolds with 21 days of TGF-β1 induction, and their enhancement were 2.6-fold, 5.2-fold, and 8.4-fold, respectively. The upregulation of these chondrogenesis specific genes was much more pronounced in 21-day chondrogenesis culture than that in 14-day chondrogenesis culture. Importantly, higher expression of chondrogenesis markers were observed in the PUS/CM culture group in contrast to that of TCF/CM group. The enhancement was 60.5-fold, 17.5-fold, 4.7-fold, 17.5-fold and 159.5-fold for ACAN, COL2A1, COL1A1, COL10A1 and SOX9, respectively.

**Fig. 10:**
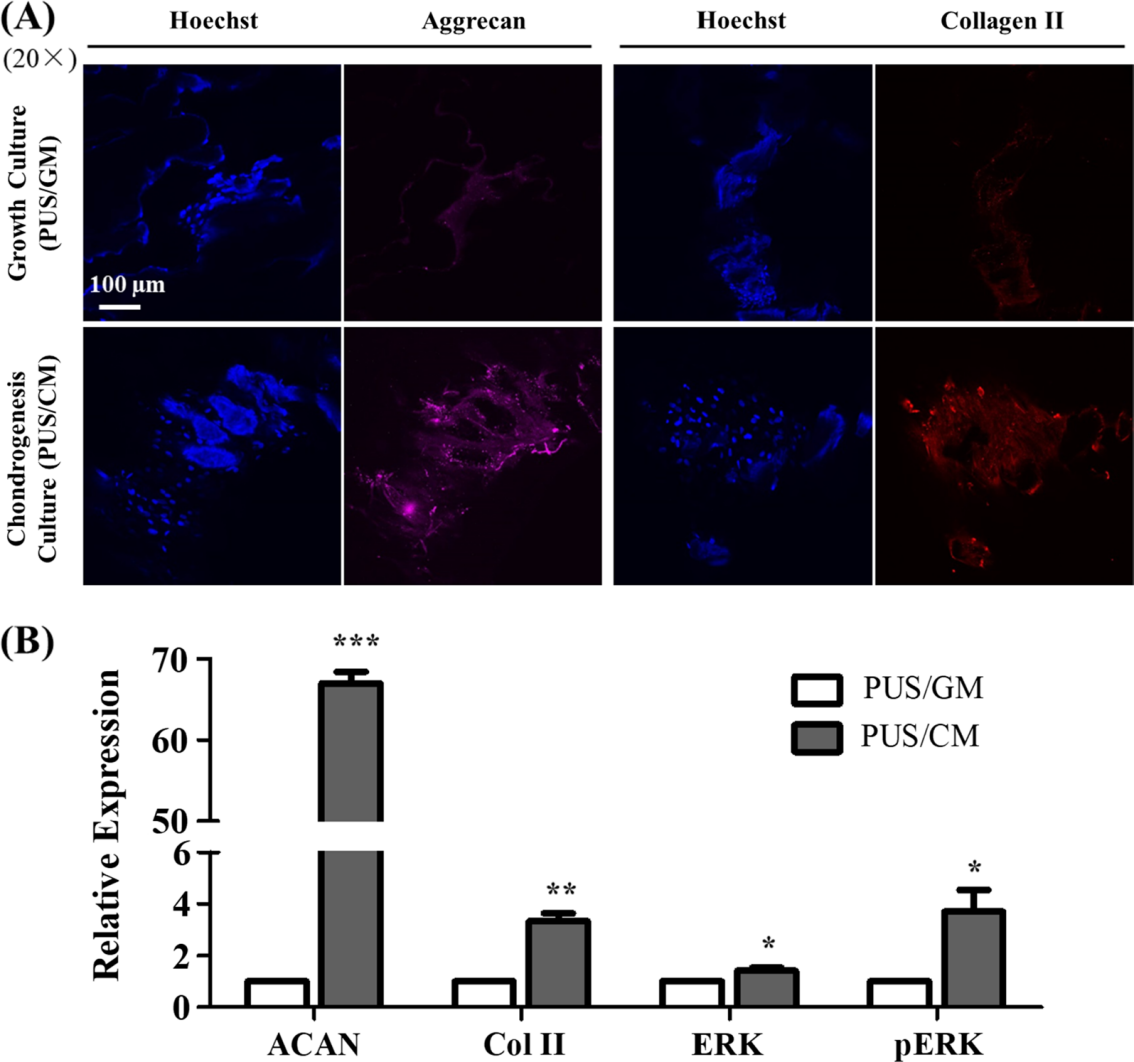
Chondrogenic differentiation of hMSCs cultured for 21 days on scaffolds assessed by **A** immunostaining of aggrecan and collagen II expression, and **B** western blot analysis of aggrecan, collagen II, ERK and pERK expression (n = 2). The relative expression values are given as the fold change compared to PUS/GM group. *p < 0.05, **p < 0.01 and ***p < 0.001

The expression and deposition of aggrecan (ACAN) and collagen II (Col II) in hMSCs on scaffolds upon chondrogenic differentiation were detected by immunofluorescence (IF) and western blot (WB). Consistent with qRT-PCR data, significantly more aggrecan and collagen II production was observed in chondrogenesis culture group than that in growth culture group in both IF and WB analysis (Fig. 10). Upon TGF-β1 induction, enhanced phosphorylation of ERK was observed in PUS/CM group compared to PUS/GM group while ERK expression stayed the similar levels based on WB analysis. The corresponding enhancement in WB analysis was 67-fold, 3.3-fold, and 3.7-fold for ACAN, Col II and pERK, respectively. The chondrogenesis can also be visualized in the 4th panel of Fig. 1, where white cartilage tissue successfully formed after 21 days of chondrogenesis culture.

## Discussion

This study describes a 3D printing-based strategy to obtain customized meniscus-like tissue using 3D porous polyurethane scaffolds in combination with hMSCs, providing insights for further development for meniscus replacement. The scaffolds were designed based on the geometry of native meniscus with choice of material and adjusted porosity so that the fabricated scaffolds recapitulated the architecture and mechanical properties of the native tissue. The use of bioactive scaffolds incorporated with hMSCs for tissue engineering addresses the limitation of poor proliferation property of chondrocytes and its difficulty in tissue formation. Therefore, the scaffolds should not only provide the mechanical support for load bearing and shock absorption, but also support cell proliferation and chondrogenic differentiation for neomeniscus regeneration. Designed porous structure coupled with appropriate surface modification are expected to enhance scaffold bioactivity by facilitation of nutrient supply, cell adhesion, cell spreading and chondrogenic differentiation.

Our fabrication approach utilizing cryo-printing technology allows precision printing and accurate tuning of pore size and porosity, which leads to tailored architecture and desired mechanical property for specific tissue. 3D porous PU meniscus scaffolds with a range of porosity were fabricated and the influence of porosity changes on mechanical property was studied (Fig. 2). The PU scaffold with 25% porosity was chosen as its mechanical property was similar to the native meniscus based on their compressive modulus values [46]. In general, these data are consistent with acknowledged trends between compressive properties and porosity, i.e., low porosity scaffolds tend to have higher compressive moduli than high porosity scaffolds [47]. It was reported that large pore/channel scaffolds with the pore size between 200 μm and 500 μm favored cell morphology maintaining, chondrocytes proliferation and ECM production [26, 48, 49]. According to the scaffold morphology obtained by SEM, our designed PU scaffolds with 25% porosity composed of micropores (1–10 µm in diameter) and macropores (200–500 µm in diameter) are anticipated to facilitate hMSCs proliferation and chondrogenic differentiation for tissue regeneration.

At present, the materials used for meniscus tissue replacement are very limited, mainly involving biodegradable materials as acellular scaffolds. The main problem of these scaffolds is that their mechanical properties and poor regenerative capability cannot satisfy the requirements of meniscus replacement in clinic. For example, collagen scaffolds degrade and shrink after being implanted in the human body, resulting in a decrease in biomechanical properties. Regarding the material selection, polyurethane and the composite material of polycaprolactone and polylactic acid (PCL/PLA) were tested. The printed PCL/PLA scaffolds displayed great hardness and brittleness, which was apparently not suitable as a meniscus implant. Polyurethane-based material was chosen for its good elasticity and abrasion resistance, and the fabricated porous meniscus-like scaffold with 25% porosity matched the mechanical properties of native meniscus. Moreover, the implanted porous PU scaffolds not only did not stimulate local inflammation and fused well with surrounding tissues, but also induced tissue ingrowth and local vascularization (Fig. 4). The exhibited good in vivo biocompatibility provides further support for its potential application in tissue regeneration.

Other material characterizations were also performed on the printed PU scaffolds and fibronectin-treated scaffolds intended for cell culture (Fig. 3). FT-IR spectra revealed that typical absorption bands of PU material was observed in printed scaffolds [50]. PU scaffolds only displayed a diffraction peak at 2θ angles around 20.9°, no additional peak at ~ 11° suggested the low crystallinity of PU scaffolds, which is consistent with the soft PU materials in the literature [51]. XPS data for S2p scan confirmed fibronectin was absorbed on the PU scaffolds after the soaking treatment. The hydrophilicity and surface charge of the prepared scaffolds were evaluated by the contact angle and zeta potential detection. The contact angles of water for PU scaffolds were reduced from 104.9° to 91.6° after FN treatment, which indicated FN absorption slightly increased its surface wettability and hydrophilicity. Relatively slight differences in the zeta potential of both types of scaffolds suggested the FN coating did not significantly change the scaffold surface charge. Therefore, the key factor for cell adhesion on the scaffolds is probably not the surface charge but the absorbed fibronectin which drives cell adhesion and subsequent functions through its interaction between the α5β1 integrin and the FN [52]. The thermal decomposition and stability of PU scaffolds matched the reported data [51]. When treated with fibronectin solution, more water was absorbed into the scaffolds, which facilitated the sitting of the scaffolds on the plate bottom for cell seeding. In addition, water-soluble matrix or absorbent material was reported to promote cell proliferation and differentiation [53, 54]. Therefore, a polymer scaffold designed in porous structure bears its superiority for tissue regeneration from this point of view.

Cell seeded polymer scaffolds usually perform better than acellular scaffolds in terms of regenerative capacity. Scaffold surface modification plays a critical role in regulating polymer bioactivity, such as in cell adhesion, proliferation, migration and differentiation [55]. Figures 5, 6, 7 revealed that hMSCs on FN-treated scaffolds maintained its spindle shape and distributed more evenly, and proliferated faster than COL-treated or PBS-treated group in tested period of time. More importantly, hMSCs on FN-treated scaffolds can migrate along the pores and grow well both on the surfaces and in the interior of the scaffold (Fig. 6). Scaffold surface modification using FN as low concentration as 50 µg/mL produced better surface property for cell function. This is consistent with the multifunctional role of FN as a master organizer of the matrix [56], and the studies using FN for scaffold treatment [37, 38, 57]. Cells grown in the interior displayed similar morphology and viability as cells on scaffold surfaces at day 14, suggesting long-term cell culture of hMSCs on porous PU scaffolds is feasible. The capability of hMSCs to proliferate on artificial scaffolds both on the surface and in the interior warrants its further development for tissue engineering.

The knee meniscus consists of water, meniscus cells, and a combination of type I and type II collagen fibers and proteoglycans in ECMs. Collagen fibers comprise the majority of the organic matter in meniscus, while aggrecan is the major large proteoglycan in meniscus [2]. Therefore, collagen and aggrecan are considered as two common chondrogenesis specific markers [33, 41, 43, 58]. It is known that TGF-β induces chondrogenic differentiation of MSCs through activation of MAPK signaling pathway including ERK/p38/JNK and their interaction with TGF‐β/Smads signaling pathway, and cartilage-specific genes and chondrogenic regulators, such as SOX9, collagen II and aggrecan, were upregulated by TGF-β [59–61]. Chondrogenic differentiation of hMSCs on this porous scaffolds upon TGF-β1 induction for 2–3 weeks was confirmed by the elevated expression of common chondrogenesis markers ACAN and COL2A1 (Figs. 8, 9). However, continued elevation of common chondrogenesis markers and upregulation of additional markers including COL1A1, COL10A1 and SOX9 was not observed until 3 weeks culture in chondrogenesis media, suggesting 2 weeks of chondrogenesis culture was not enough for sufficient induction of a more mature differentiation state. The observed time-dependent chondrogenesis states is in line with SOX9 time-course expression profile. Compared with the corresponding growth culture group, SOX9 expression decreased by ~ twofold in 14 days of chondrogenesis culture, but increased by ~ eightfold in 21 days of chondrogenesis culture. It is well established that SOX9 controls cell lineage fate and is required for chondrogenesis differentiation, therefore it has been recognized as the master regulator of chondrogenesis [62–64]. As such, SOX9 upregulation can serve as a critical indicator of true chondrogenesis [64]. In addition, significant elevation of cartilage-related gene expression by hMSCs in scaffold/chondrogenesis culture group in contrast to that in TC flask/chondrogenesis culture group suggested porous PU scaffolds coupled with surface modification greatly facilitated chondrogenesis differentiation of hMSCs. Polymer’s intrinsic weakness of low bioactivity in tissue engineering can be counteracted by surface modification, and FN appears to be an optimal option. Increased production of aggrecan and collagen II observed from immunostaining and WB (Fig. 10) was consistent with the dramatic gene upregulation of ACAN and COL2A1 obtained from qRT-PCR analysis. Activation of ERK signaling pathway upon TGF-β1 induction was confirmed by enhanced pERK expression (Fig. 10B). All of these data proved that hMSCs seeded on the porous PU scaffolds have been successfully differentiated into chondrocyte-like cells. In situ long-term cell culture and chondrogenesis of 3D porous PU meniscus scaffolds by hMSCs upon induction demonstrates its potential value in cartilage engineering and meniscus replacement.

Mesenchymal stem cells, neural stem cells or induced pluripotent stem cells (iPSCs) can transdifferentiate or differentiate to neural precursors and/or mature neurons, or glial cells. Combined with stem cells, multiple types of scaffolds have been investigated for neurogenesis, including natural components such as alginate [65] and native ECM [66, 67], and synthetic material such as polymers [68, 69] and graphene [70, 71]. This sheds light on the possibility of using scaffolds in combination with MSCs to replace damaged neurons. Based on the performance of the prepared porous scaffolds in this study, it is reasonable to anticipate that the prepared scaffolds should also facilitate neuronal differentiation under appropriate culture and induction conditions, and may serve as a promising substrate for neuronal regeneration. Coupled with proper surface treatment, the precisely fabricated porous scaffolds possess high bioactivity, which opens up its possibility in other tissue regeneration application.

## Conclusion

In this study, 3D printing-based strategy was employed to fabricate customized meniscus scaffolds, and such scaffolds populated with high number of hMSCs committed with chondrogenic lineage demonstrates its potential regeneration value in damaged meniscus treatment. These scaffolds can be precisely tailored to mimic the specific architecture and favorable mechanical property of native meniscus. The porous structure coupled with surface modification of the meniscus scaffold greatly facilitates long-term hMSC culture and stem cell function including cell adhesion, proliferation, migration and differentiation. In situ deep chondrogenesis of 3D porous PU scaffolds by hMSCs proved by SOX9 and ECM upregulation highlights its efficacy in cartilage tissue regeneration. Although there is still a long way to generate a fully functional meniscus construct, the use of 3D porous PU scaffolds incorporated with hMSCs deserves further investigation to evaluate its in vivo performance and long-term preservation of meniscus function.

## Data Availability

The data that support the findings of this study are available on request from the corresponding author.
